# Clinical Relevance of Vaginal Cuff Dehiscence after Minimally Invasive versus Open Hysterectomy

**DOI:** 10.3390/jcm12083001

**Published:** 2023-04-20

**Authors:** Kyung Jin Eoh, Young Joo Lee, Eun Ji Nam, Hye In Jung, Young Tae Kim

**Affiliations:** 1 Department of Obstetrics and Gynecology, Yongin Severance Hospital, Yonsei University College of Medicine, Yongin 16995, Republic of Korea; kjeoh2030@yuhs.ac; 2 Department of Obstetrics and Gynecology, Institute of Women’s Medical Life Science, Yonsei Cancer Center, Severance Hospital, Yonsei University College of Medicine, Seoul 03722, Republic of Korea; leeyj_0907@yuhs.ac (Y.J.L.); nahmej6@yuhs.ac (E.J.N.);

**Keywords:** hysterectomy, minimally invasive surgical procedure, dehiscence, complication

## Abstract

This study aimed to evaluate the clinical relevance of vaginal cuff dehiscence following a hysterectomy. Data were prospectively collected from all patients who underwent hysterectomies at a tertiary academic medical center between 2014 and 2018. The incidence and clinical factors of vaginal cuff dehiscence after minimally invasive versus open hysterectomy were compared. Vaginal cuff dehiscence occurred in 1.0% (95% confidence interval [95% CI], 0.7–1.3%) of women who underwent either form of hysterectomy. Among those who underwent open (n = 1458), laparoscopic (n = 3191), and robot-assisted (n = 423) hysterectomies, vaginal cuff dehiscence occurred in 15 (1.0%), 33 (1.0%), and 3 (0.7%) cases, respectively. No significant differences in cuff dehiscence occurrence were identified in patients who underwent various modes of hysterectomies. A multivariate logistic regression model was created using the variables indication for surgery and body mass index. Both variables were identified as independent risk factors for vaginal cuff dehiscence (odds ratio [OR]: 2.74; 95% CI, 1.51–4.98 and OR: 2.20; 95% CI, 1.09–4.41, respectively). The incidence of vaginal cuff dehiscence was exceedingly low in patients who underwent various modes of hysterectomies. The risk of cuff dehiscence was predominantly influenced by surgical indications and obesity. Thus, the different modes of hysterectomy do not influence the risk of vaginal cuff dehiscence.

## 1. Introduction

Hysterectomy is a commonly performed surgical procedure within the field of gynecology, that is conducted globally. However, akin to any surgical intervention, it presents a range of potential complications, including but not limited to hemorrhage, urinary tract injury, gastrointestinal injury, postoperative fever, and vaginal vault prolapse [1,2,3]. Although rare, patients may also experience vaginal cuff dehiscence following the surgery, either with or without bowel evisceration.

Variations in diagnostic criteria, the approach to colpotomy, and differences in cuff closure techniques and suture materials employed may contribute to the variability in the documented incidence of vaginal cuff dehiscence. The literature indicates that the occurrence of vaginal cuff dehiscence is relatively infrequent, with reported rates ranging from 0.2% to 4.8% in women undergoing abdominal hysterectomy, and 0.8% to 3.7% in women undergoing total laparoscopic hysterectomy [4,5,6,7,8,9,10]. Nonetheless, recent meta-analytical evidence suggests that the utilization of laparoscopic closure for the vaginal vault and barbed sutures may reduce the incidence of vaginal cuff dehiscence [11].

Previous studies have reported a range of 0.4% to 4.1% incidence rates of vaginal cuff dehiscence among women who undergo robotic-assisted hysterectomy [12,13,14]. It is noteworthy that patients undergoing minimally invasive surgical procedures, including robotic-assisted hysterectomy, exhibit a greater risk of vaginal cuff dehiscence compared to those undergoing open procedures. Kho et al. have hypothesized that this augmented risk may stem from thermal damage to the vaginal area during colpotomy [14]. Nevertheless, the data on the incidence of vaginal cuff dehiscence following robotic-assisted hysterectomy remain limited. The utilization of robotic wristed instruments may provide greater flexibility in the extent of vaginal tissue dissection, potentially impacting the likelihood of dehiscence. For instance, more specialized suturing of the vaginal vault may be feasible, which could potentially reduce the risk of postoperative vaginal cuff dehiscence.

Over the past two decades, laparoscopic and robotic hysterectomies have become increasingly popular in gynecologic surgery due to their advantages over traditional open surgery. Various techniques have been suggested to reduce the risk of vaginal cuff dehiscence after endoscopic hysterectomies, such as delaying the resumption of sexual activity after surgery, minimizing the use of electroenergy, reinforcing closures with double layers or additional materials, providing adequate training for endoscopic suturing, employing barbed sutures, and using a transvaginal approach for cuff closure. However, despite the significance of this issue, there is still no agreement or established guidelines regarding the most effective methods for preventing vaginal cuff dehiscence.

Our study aimed to assess the occurrence of vaginal cuff dehiscence and identify its risk factors. We analyzed prospectively collected medical data for a consecutive cohort of patients who underwent various types of hysterectomy, including open, conventional laparoscopic, and robot-assisted procedures.

## 2. Materials and Methods

The present study was subjected to ethical review and obtained approval from the Institutional Review Board (IRB) of Yonsei University in Korea (approval code 4-2020-1369). Given that the study used retrospectively collected medical data, the requirement for written informed consent was waived by the IRB. This is in accordance with the guidelines set forth by the Declaration of Helsinki, which recognizes that in certain circumstances, such as retrospective medical record reviews, obtaining informed consent may not be practical or feasible. Nonetheless, the study ensured that the privacy and confidentiality of the patients’ medical information were protected, and no identifiable information was disclosed in the study report.

**Figure 1:**
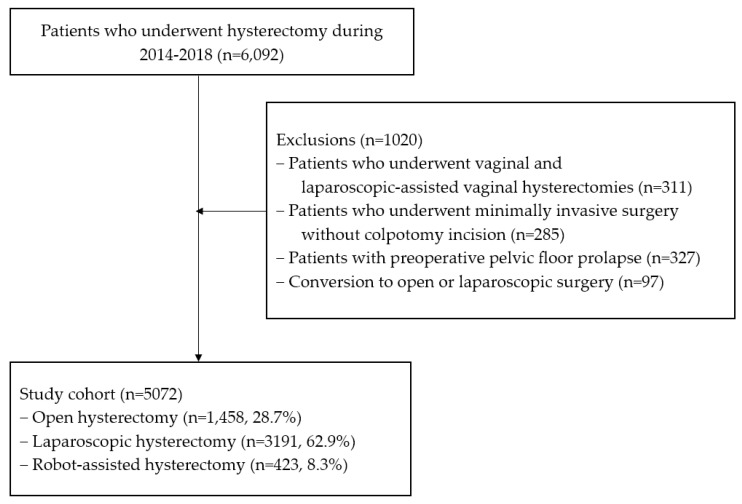
Flow diagram of the study cohort.

All medical records of patients who underwent open, total laparoscopic, or robotic hysterectomy for benign or malignant diseases between January 2014 and December 2018 were collected for analysis (as shown in Figure 1). Patients who underwent conversion to an open procedure from minimally invasive procedures or conventional laparoscopic-assisted hysterectomy from robot-assisted surgery were excluded from the analysis. Additionally, we excluded patients who underwent vaginal and laparoscopic-assisted vaginal hysterectomies because these surgeries did not involve a peritoneal approach for colpotomy incision. Patients who underwent minimally invasive surgery without colpotomy incision were excluded from the analysis as well. Patients with preoperative pelvic floor prolapse were excluded from the study.

**Figure 2:**
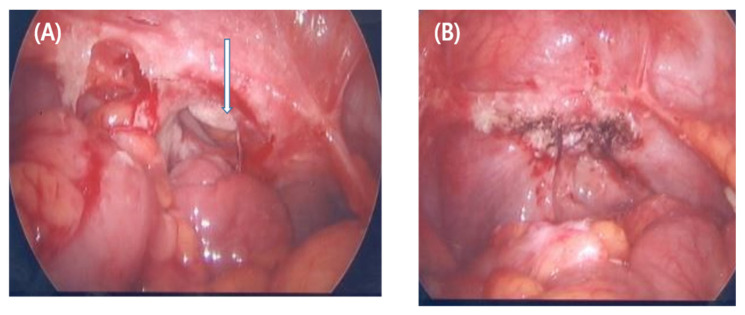
(**A**) The vaginal cuff shows complete dehiscence. White gauze can be observed in the upper vagina (arrow). (**B**) The vaginal cuff dehiscence closed laparoscopically using a barbed suture (V-Loc).

To ensure that only patients who developed vaginal cuff dehiscence as a complication of hysterectomy were included in the study (as shown in Figure 2), the medical records of patients who developed dehiscence were thoroughly reviewed. The study collected data for each patient, including age, menopausal status, parity, body mass index (BMI), indication for initial hysterectomy, administration of prophylactic antibiotics, and whether or not a transfusion was required. Vaginal cuff dehiscence was defined as a full-thickness opening of the anterior and posterior edges of the vaginal cuff without protruding bowel. The surgical procedures performed, including open and laparoscopic hysterectomies, followed previously described techniques [15,16,17,18,19,20].

Surgical techniques for hysterectomy vary widely and can impact the incidence of vaginal cuff dehiscence. In this study, both open and minimally invasive hysterectomies were performed, with the colpotomy incision made at the vaginal fornices using either a scalpel or a monopolar hook/spatula set at 60 W with a pure cutting waveform. The choice of closure method was left up to the discretion of the surgeon, which could have led to variability in the technique used. Laparoscopic hysterectomies involved closing the vaginal cuff with interrupted sutures of polyglactin 0, secured with either a laparoscopic knot pusher or sutures of polyglactin 2-0. In contrast, robotic hysterectomy cases utilized the da Vinci Surgical System, which allowed for more precise control and visualization. The colpotomy was made using monopolar scissors (35 W, blended mode), and the vaginal cuff was reapproximated using 0 Vicryl suture placed in a figure-of-eight pattern with intracorporeal knot-tying or with 0 Vicryl suture using the Endo Stitch device with extracorporeal knot-tying, based on the surgeon’s preference.

All statistical analyses were conducted using SPSS 26 software (IBM Corp., Chicago, IL, USA). Prior to conducting the analyses, the normality of data distribution was carefully assessed using the Shapiro-Wilk test to ensure that appropriate statistical tests were utilized. Continuous variables were compared using the Student’s *t*-test, or the non-parametric Mann-Whitney test was used when normal distribution could not be assumed. Proportions were compared using Fisher’s exact test, which is a powerful tool for testing the significance of differences between groups in small sample sizes. To analyze the risk factors associated with vaginal cuff dehiscence, univariate logistic regression was performed to assess the statistical significance of individual variables. The variables found to be statistically significant were included in a multivariable logistic regression model, which allowed for a more nuanced understanding of the relationships between variables. By calculating the odds ratios (ORs) and 95% confidence intervals (CIs) using this model, the present study was able to identify the specific risk factors that were most strongly associated with vaginal cuff dehiscence.

## 3. Results

During the 5-year study period, a total of 5072 hysterectomies were included in the present study. Of these, 1458 (28.7%) were open hysterectomies, 3191 (62.9%) were laparoscopic hysterectomies, and 423 (8.3%) were robotic hysterectomies. The study found a total of 51 cases of vaginal cuff dehiscence, with 15 (1.0%; 95% confidence interval [CI], 0.5–1.6%) occurring in women who underwent an open hysterectomy, 33 (1.0%; 95% CI, 0.7–1.4%) in women who underwent a total laparoscopic hysterectomy, and 3 (0.7%; 95% CI, 0.0–1.6%) in women who underwent a robotic hysterectomy. The overall incidence of vaginal cuff dehiscence was 1.0% (95% CI, 0.7–1.3%) in the total cohort, indicating that the risk of vaginal cuff dehiscence is similar across different types of hysterectomy. Table 1 shows the total number of cases and vaginal cuff dehiscence per year, which remained relatively stable over the 5-year study period. Fisher’s exact test indicated that there was no significant difference in the incidence of dehiscence between 2014 and 2018 (*p* = 0.455), suggesting that the risk of vaginal cuff dehiscence did not increase over time.

Table 2 presents a comprehensive comparison of the clinical characteristics of patients who experienced vaginal cuff dehiscence and those who did not. The study included patients who underwent hysterectomy for either benign or malignant gynecologic surgical indications. Out of the total cohort, 515 patients (10.2%) had cervical cancer, 482 patients (9.5%) had endometrial cancer, 229 patients (4.5%) had ovarian and tubal cancer, and 3846 patients (75.8%) had benign diseases, with symptomatic uterine leiomyoma being the most common indication for hysterectomy. Interestingly, the incidence of cuff dehiscence was observed to be higher in patients with malignant indications as compared to those with benign indications, with a statistically significant *p*-value of 0.012. However, age, gravidity, menopausal status, preoperative hemoglobin level, presence of intraoperative transfusion, and mode of surgery were not found to be statistically significantly different between patients who experienced vaginal cuff dehiscence and those who did not, as per Table 2.

The univariate logistic regression analysis was carried out to evaluate the significance of several individual variables as predictors of vaginal cuff dehiscence, including age, BMI, indication for surgery, gravida, menopausal state, presence of intraoperative transfusion, and mode of surgery. Out of these variables, it was found that only the indication for surgery and BMI had a statistically significant association with cuff dehiscence. We proceeded to develop a multivariable logistic regression model, which incorporated the two significant variables identified in the previous analysis, i.e., indication for surgery and BMI. The application of this model confirmed that the indication for surgery and BMI were independently associated with an increased risk of vaginal cuff dehiscence (odds ratio [OR]: 2.74; 95% CI, [1.51–4.98] and OR: 2.20; 95% CI, [1.09–4.41], respectively) (Table 3).

## 4. Discussion

The current investigation aimed to assess the incidence of vaginal cuff dehiscence and identify its associated risk factors in patients undergoing hysterectomy through various surgical methods, including open, laparoscopic, and robotic techniques. The study sought to determine whether a difference existed in the occurrence of vaginal cuff dehiscence among these surgical methods. Our results revealed that the incidence of vaginal cuff dehiscence was minimal across all patients who underwent any of the three surgical approaches. Additionally, no significant differences were found in the occurrence of cuff dehiscence among patients undergoing open, laparoscopic, or robotic hysterectomy. To the best of our knowledge, this is the first study to investigate the incidence and risk factors of vaginal cuff dehiscence in consecutive patient cohorts who underwent all three types of hysterectomy.

Vaginal cuff dehiscence is a rare but reported complication of hysterectomy, with a wide range of reported incidence rates. Variations in the diagnostic method used to define dehiscence, the mode of hysterectomy, the colpotomy technique, and different methods of vaginal cuff closure could account for the variations in the reported rates of occurrence. In the present study, the incidence of vaginal cuff dehiscence was 1.0%, which is consistent with the incidence rates reported in the literature [4,5,6,7,8,21,22,23,24]. Prior studies have indicated that vaginal cuff dehiscence occurs in 0.2–4.8% of women who undergo abdominal hysterectomy, 0.8–3.7% of women undergoing total laparoscopic hysterectomy, and 0.4–4.1% of women undergoing robotic-assisted hysterectomy [12,13,14,25,26,27]. Some studies have suggested that minimally invasive approaches, including the robotic platform, are associated with an increased risk of vaginal cuff separation [5,8,9,14,28,29,30]. However, recent meta-analyses have reported that laparoscopic closure of the vaginal vault and the use of barbed sutures may decrease the rate of vaginal cuff separation [11].

In previous research, it has been observed that more than half of the cases of vaginal cuff dehiscence were related to sexual activity. Nevertheless, no study has yet examined the recommended duration of sexual abstinence after an endoscopic hysterectomy. Furthermore, while coital activity is commonly associated with vaginal cuff dehiscence, some cases have been reported without any identifiable trigger or as spontaneous occurrences. A randomized trial conducted by Uccella et al. investigated potential factors that contribute to vaginal cuff dehiscence and discovered that smoking was associated with an increased risk, while obesity and postmenopausal status had a protective effect against this complication [31].

The available evidence is limited, but a systematic review previously reported that laparoscopic cuff closure is associated with a lower risk of vaginal cuff dehiscence compared to transvaginal cuff closure. Similarly, the use of barbed sutures has been shown to decrease the risk of dehiscence compared to nonbarbed sutures, mainly polyglactin. Self-anchoring sutures, made of a delayed-absorbable material, prevent tissue slippage and eliminate the need to tie knots during endoscopic surgery [11,32]. It is important to approach the recommendation for the use of barbed sutures with caution due to several reasons. Firstly, the meta-analysis is mainly influenced by a single retrospective study by Siedhoff et al., where the incidence of dehiscence was 0% vs. 4.4% in the barbed vs control groups, respectively. The high incidence of separations in the control group raises questions about the quality of closures performed [33]. Secondly, the control group in the study used several different techniques and approaches for vaginal suture, including monofilament, braided, and coated sutures, Endostitch, and robotic closure, which is a well-known risk factor for vaginal dehiscence [33]. Thirdly, when considering only studies on total laparoscopic hysterectomies (excluding robotic cases), the risk of dehiscence is not significantly different between barbed and conventional braided and coated sutures. This raises questions about whether the difference could be attributed to the surgical approach rather than the suture material used [34]. It is also worth noting that self-anchoring sutures have been associated with rare but severe complications, such as bowel obstruction [35]. Therefore, more high-level evidence based on larger patient series is necessary before strong recommendations can be made in support of their use for preventing post-hysterectomy vaginal cuff separations.

While the traditional approach to total laparoscopic hysterectomy involves performing each surgical step, including suturing of the vault, laparoscopically, some authors have reported successful outcomes with a transvaginal approach [8,34,36]. However, a previous meta-analysis found that transvaginal closure of the cuff significantly increases the risk of vaginal dehiscence. This evidence is mainly based on a single randomized trial of 1408 patients, which aimed to show that transvaginal suturing is superior to laparoscopic closure, contrary to the findings of previous retrospective studies. Nevertheless, the results of the trial supported the opposite conclusion. Further studies are required to confirm this recommendation. Possible explanations have been proposed for the lower incidence of dehiscence in patients who received laparoscopic suturing of the vault, such as the inclusion of the peritoneum of the pouch of Douglas, which may help reduce postoperative bleeding and inflammation in the vaginal submucosa. Additionally, technological improvements in cameras and laparoscopes may enhance the surgeon’s visual precision during suturing. Finally, the transvaginal approach may be challenging and less consistent than laparoscopic closure in some cases, such as when the vagina is long or narrow.

The frequent utilization of electrosurgery has been traditionally linked to the increased risk of vaginal breakdowns after laparoscopic hysterectomy when compared to abdominal and vaginal hysterectomy [37]. However, a prior meta-analysis failed to establish any evidence of the role of electroenergy or other forms of energy in the development of vaginal cuff dehiscence. This study also found no data supporting the use of reinforced or double-layer sutures in comparison to the standard single-layer method. Similarly, no research has demonstrated the advantages of comprehensive training in vaginal cuff suturing in decreasing the risk of vaginal cuff dehiscence. Nevertheless, the meta-analysis suggests that extensive training to enhance the laparoscopic suturing technique should be prioritized to decrease the incidence of this complication, since a deficient knot-tying technique may potentially be associated with a higher likelihood of vault complications.

To evaluate the relationship between vaginal cuff dehiscence and the mode of hysterectomy, a large randomized controlled trial (RCT) would be the most appropriate study design. However, due to the rarity of this complication, previous RCTs that compared different hysterectomy approaches did not have sufficient statistical power to determine a clinically significant difference in cuff dehiscence. Therefore, available data on this topic come from case series and cohort studies. For example, Iaco et al. conducted a study that compared vaginal cuff dehiscence and evisceration rates after different modes of hysterectomy, including laparoscopic hysterectomy, and concluded that the surgical route did not influence the risk of dehiscence [38]. Moreover, with the improvement and standardization of minimally invasive hysterectomy techniques, a low incidence of vaginal cuff dehiscence has been maintained.

The current study design, although nonrandomized, enabled us to control some of the variations in cuff dehiscence rates that arise from demographic factors, obesity, and surgical procedures. This allowed us to investigate various clinical and surgical parameters as risk factors for vaginal cuff dehiscence. A multivariable logistic regression model was used to determine the likelihood of developing vaginal cuff dehiscence in patients with malignant surgical indications, which was found to be 2.7-fold higher than in cases with benign indications. Patients with malignant diseases who undergo total hysterectomy require more intensive care, as they often have malnutrition and multiple comorbidities that may contribute to the risk of vaginal cuff dehiscence. Ceccaroni et al. also reported malignancy as a significant independent risk factor for vaginal cuff dehiscence, with an incidence of 0.8% after total hysterectomy for malignant indications, compared to 0.2% after total hysterectomy for benign indications [39].

Drudi et al. conducted a study on a group of patients who received postoperative adjuvant therapy, which showed a higher incidence of vaginal cuff dehiscence of 3% compared to 0.4% in patients who did not receive such treatment. The authors noted that the use of adjuvant chemotherapy can interfere with wound healing and compromise the structural integrity of the vaginal cuff, making it a confounding factor in several studies on vaginal cuff dehiscence. In cases of locally advanced cervical cancer treated with primary chemoradiation, there is up to a 50% incidence of residual disease, which directly increases the risk of local recurrence. Completion surgery in these patients poses an additional risk of vaginal cuff dehiscence with evisceration due to the poor tissue quality of the vaginal cuff [24].

The present study found a significant association between vaginal cuff dehiscence and obesity. Patients with a BMI greater than 25 kg/m^2^ had a 2.2-fold higher risk of developing vaginal cuff dehiscence compared to those with a BMI less than 25 kg/m^2^. BMI is the most common measure of obesity and is calculated as weight in kilograms divided by height in meters (kg/m^2^). While the definition of obesity depends on the method used, we defined obesity based on the Korea National Health and Nutrition classification of BMI [40]. Obesity is associated with poor wound healing and worse clinical comorbidities, such as diabetes, hypertension, and cancer [41,42,43,44]. The condition of obesity leads to the development of an inflammatory environment in the body, characterized by elevated levels of various markers such as C-reactive protein, interleukin-6, and tumor necrosis factor-α in the bloodstream. Moreover, there is a deficiency of immune cells that are essential for wound protection and healing, which collectively affects the wound healing mechanism and possibly worsens its clinical implications [45,46].

The study conducted on vaginal cuff dehiscence after hysterectomy had a number of strengths. One of the most significant strengths of the study was the large sample size that was achieved. This was possible due to the long-term study period, which allowed for a significant number of patients to be included in the study. The large sample size was important as it enabled the researchers to accurately analyze the low incidence rate of vaginal cuff dehiscence after hysterectomy. Another strength of the study was that it included various types of surgical approaches, including open and minimally invasive procedures, such as robotic assistance. This added power to the results as it allowed for a more comprehensive analysis of the incidence rate of vaginal cuff dehiscence across different surgical approaches. Additionally, all surgeries were performed by experienced gynecologic oncologists and designated gynecologists at a tertiary referral institution. This ensured that the surgeries were performed by qualified professionals, minimizing the risk of complications, and ensuring the accuracy of the results.

However, despite the strengths of the study, there are some limitations that need to be considered. Firstly, the study had a retrospective design, which may have led to unmeasured variables that could cause confounding. This means that the researchers may not have been able to control for all potential confounding factors, which could have influenced the results. Secondly, different patient and surgeon variables may have influenced the choice of surgical approach for a hysterectomy. For example, patient factors such as age, medical history, and comorbidities, as well as surgeon factors such as experience and preference, could have influenced the choice of surgical approach. This means that the results of the study may not be generalizable to all patient populations or surgical settings.

## 5. Conclusions

In summary, our findings indicate that the risk of vaginal cuff dehiscence following hysterectomy is not increased with the use of minimally invasive surgical methods, such as laparoscopy and robot-assisted surgery, even when factoring in the learning curve for gynecologic surgeons. Considering the rarity of this complication, a multi-center prospective study investigating outcomes following a hysterectomy is necessary.

## Figures and Tables

**Table 1:** Number of cases of hysterectomies involving colpotomy and vaginal cuff dehiscence in each year of the study.

Year	No. of Total Cases	No. of Vaginal Cuff Dehiscence	Incidence (%)
			(95% CI)
2014	844	10	1.2 (0.5–1.9)
2015	923	13	1.4 (0.7–2.2)
2016	1090	7	0.6 (0.2–1.1)
2017	1071	12	1.1 (0.6–1.8)
2018	1093	9	0.8 (0.4–1.4)
Total	5072	51	1.0 (0.7–1.3)

CI, confidence interval.

**Table 2:** Patient characteristics of the study population according to the presence of vaginal cuff dehiscence.

	Overall	No Dehiscence	Dehiscence	
	(n = 5072)	(n = 5021)	(n = 51)	*p*
Age				
<50 years	3204 (63.2)	3167 (63.1)	37 (72.5)	0.19
≥50 years	1868 (36.8)	1854 (36.9)	14 (27.5)	
BMI				
<25 kg/m^2^	1756 (34.6)	1746 (34.8)	10 (19.6)	0.024
≥25 kg/m^2^	3316 (65.4)	3275 (65.2)	41 (80.4)	
Indication (%)				
Benign disease	3846 (75.8)	3815 (76.0)	20 (39.2)	0.012
Malignancy	1226 (24.2)	1206 (24.0)	31 (60.8)	
Gravida (%)				
0	524 (10.3)	520 (10.4)	4 (7.8)	0.557
≥1	4548 (89.7)	4501 (89.6)	47 (92.2)	
Menopause (%)				
Yes	1869 (36.8)	1855 (36.9)	14 (27.5)	0.162
No	3203 (63.2)	3166 (63.1)	37 (72.5)	
Preop Hb (SD)	12.37 (1.68)	12.39 (1.69)	11.81 (1.71)	0.874
Transfusion (%)				
Yes	134 (2.6)	133 (2.6)	1 (2.0)	0.76
No	4938 (97.4)	4888 (97.4)	50 (98.0)	
Mode of surgery (%)				
Open	1458 (28.7)	1443 (8.7)	15 (29.4)	0.816
Laparoscopic	3191 (62.9)	3158 (62.9)	33 (64.7)	
Robotic	423 (8.3)	420 (8.4)	3 (5.9)	

Data are presented as the mean (SD) or n (%). SD, standard deviation; BMI, body mass index; Preop, preoperative.

**Table 3:** Logistic regression analyses of factors influencing vaginal cuff dehiscence.

	Univariate		Multivariate	
Variable	OR (95% CI)	*p* Value	OR (95% CI)	*p* Value
Age < 50 years	1.47 (0.82–2.64)	0.197		
BMI ≥ 25 kg/m^2^	2.19 (1.09–4.37)	0.027	2.20 (1.09–4.41)	0.028
Indication				
Benign	1.00 (Reference)		1.00 (Reference)	
Malignancy	2.04 (1.16–3.59)	0.013	2.74 (1.51–4.98)	0.001
Gravida				
No	1.00 (Reference)			
Yes	0.74 (0.26–2.05)	0.559		
Menopause				
No	1.00 (Reference)			
Yes	1.55 (0.84–2.87)	0.165		
Transfusion				
No	1.00 (Reference)			
Yes	1.36 (0.19–9.92)	0.761		
Mode of surgery				
Open	1.00 (Reference)			
Laparoscopic	1.45 (0.42–5.05)	0.56		
Robotic	1.46 (0.45–4.79)	0.53		

OR, odd ratio; CI, confidence interval; BMI, body mass index.

## Data Availability

Due to the nature of this retrospective study, participants of this study did not agree for their data to be shared publicly, so supporting data is not available.
